# *Notes from the Field:* Heat-Related Emergency Department Visits — June 28–July 7, 2026

**DOI:** 10.15585/mmwr.mm7536a2

**Published:** 2026-09-17

**Authors:** Ambarish Vaidyanathan, Abigail Gates, Paul Iniguez, Jessica Lee, Aaron Bernstein

**Affiliations:** ^1^Division of Environmental Health Science and Practice, National Center for Environmental Health, CDC; ^2^Detect and Monitor Division, Office of Public Health Data, Surveillance, and Technology, CDC; ^3^Weather Prediction Center, National Weather Service, National Oceanic and Atmospheric Administration, College Park, Maryland; ^4^National Center for Environmental Health, Agency for Toxic Substances and Disease Registry, CDC.

**Summary**

**What is already known about this topic?**

Sustained periods of extreme heat are associated with increased heat-related emergency department (ED) visits and deaths; certain groups, including working-age and older adults, often experience more heat-related illnesses than do younger persons.

**What is added by this report?**

During a 10-day period of extreme heat in late June and early July 2026, heat-related ED visit rates in the eastern, midwestern, southern, and south-central regions of the United States were markedly higher than those during the same dates in earlier years; the highest rates occurred around the July 4 holiday.

**What are the implications for public health practice?**

Syndromic surveillance and early heat warning systems are critical for rapidly detecting increases in heat-related illness during extreme heat events and can guide timely public health responses, including targeted prevention efforts for populations and regions at highest risk.

## Introduction

National surveillance reports continue to document heat-related deaths and emergency department (ED) visits across the United States (*1*,*2*). Heat exposure is linked to a range of adverse health outcomes, from mild conditions including dehydration and heat exhaustion, to severe complications including heat stroke, organ failure, and death (*1*,*3*). Persons affected by heat-related illness (HRI) often have underlying comorbidities that heighten their susceptibility to heat stress, such as cardiovascular disease, respiratory conditions (e.g., asthma), kidney disease, or other chronic medical conditions (*3*). The ongoing 2026 heat season in the United States has produced some of the highest temperatures on record (*4*,*5*). In late June and early July, a heat dome, a weather pattern in which a strong, persistent high-pressure system traps hot air over an area and causes unusually high temperatures, persisted for approximately 10 days (June 28–July 7) and affected large portions of the eastern, midwestern, southern, and south-central United States.

## Investigation and Outcomes

### Identification of HRI ED Visits

To characterize changes in HRI ED visits associated with this heat dome, CDC analyzed data from the National Syndromic Surveillance Program (NSSP) by comparing daily HRI ED visits per 100,000 all-cause ED visits (visit rates) during June 28–July 7, 2026, with visit rates observed during the same calendar dates during 2018–2025. Data were extracted from NSSP’s Electronic Surveillance System for the Early Notification of Community-Based Epidemics (ESSENCE), and daily counts of HRI and all-cause ED visits were aggregated for each of the 10 U.S. Department of Health and Human Services (HHS) regions and stratified by age group and sex (*2*). To minimize the impact of temporal changes in facility participation, the study restricted comparisons across years to EDs that reported consistently during the study period.*

### Assessment of U.S. Heat Exposure Patterns

HeatRisk is a five-level, location-specific forecasting framework developed by the National Oceanic and Atmospheric Administration’s National Weather Service (NOAA/NWS) and CDC that integrates meteorologic data with human health metrics to assess and communicate the progressive, population-level risks (none/low, minor, moderate, major, and extreme) of heat exposure in real time. In collaboration with NOAA/NWS, HeatRisk data were processed and county-level maximum daily values were mapped for June 28–July 7, 2026, to assess differences in heat exposure patterns across the United States. This activity was reviewed by CDC, deemed not research, and conducted consistent with applicable federal law and CDC policy.^†^

### HRI ED Visits and HeatRisk Levels by HHS Region

As of September 10, 2026, ESSENCE recorded 145,321 HRI ED visits in 2026,^§^ approximately 15% (21,775) of which occurred in HHS regions 1–7 (primarily covering the eastern, midwestern, southern, and south-central regions of the United States) during June 28–July 7, 2026. During this time, approximately 65% of the U.S. population (221 million persons) in 42 states and the District of Columbia experienced ≥1 day of major or extreme HeatRisk (Figure). Consistent with these conditions, daily HRI visit rates in these regions substantially exceeded those during the same dates during 2018–2025. In the majority of HHS regions, daily visit rates exceeded a previously established benchmark defined as the 95th percentile of HRI visit rates (*2*) during 2018–2025; in HHS regions 2, 3, and 5, the maximum daily HRI visit rates recorded since 2018 occurred on or just before the July 4 holiday weekend. HRI ED visit rates were highest among men aged 26–54 years in HHS regions 1, 3, 4, 5, 6, and 7. In HHS Region 2, visit rates were highest among men aged ≥55 years.

**FIGURE:**
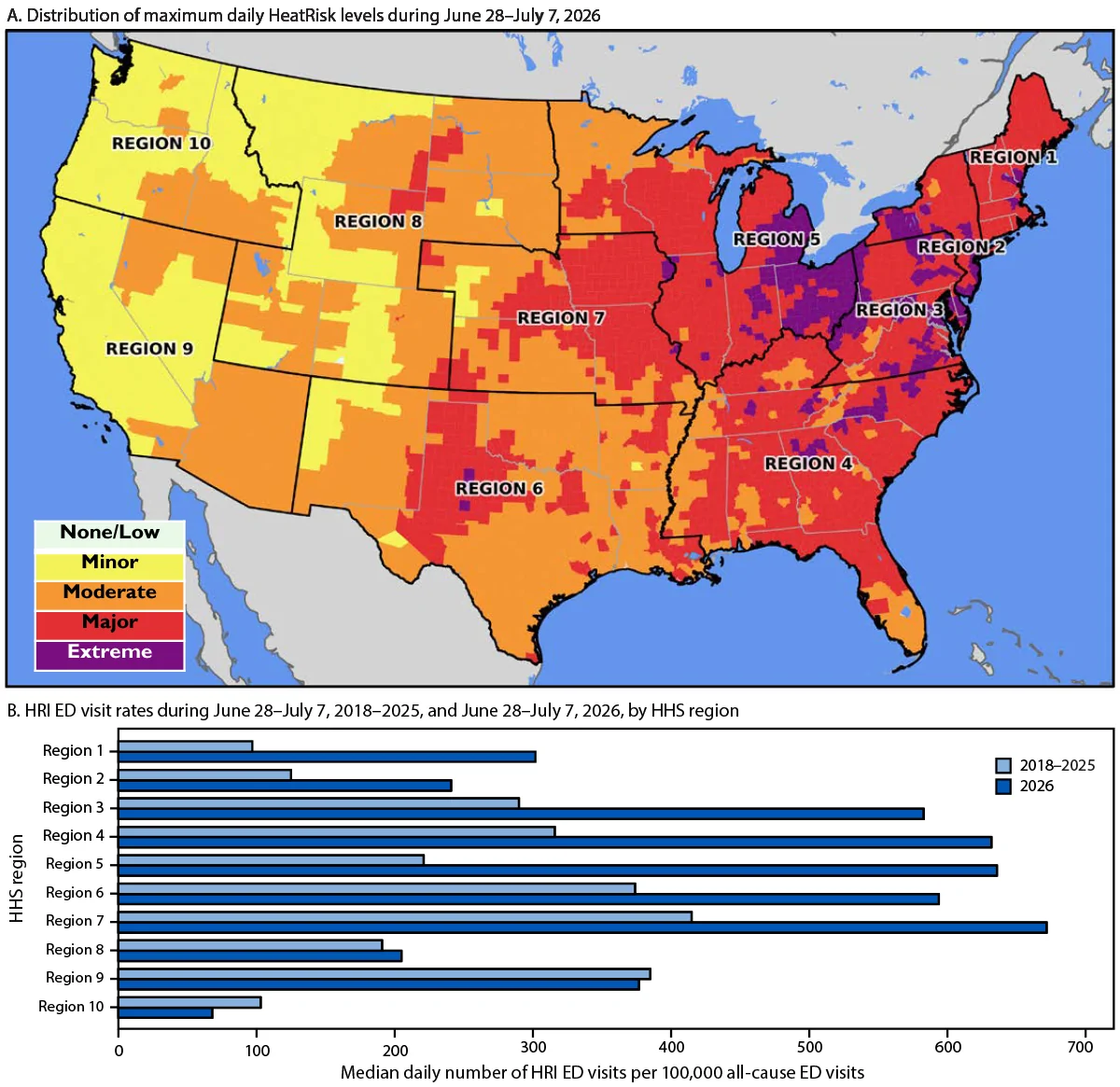
Distribution of maximum daily HeatRisk levels,* by county (A), and comparison of median daily heat-related illness emergency department visit rates,^†^ by region^§^ and period (B) — United States,^¶^ June 28–July 7, 2018–2025, and June 28–July 7, 2026 **Abbreviations:** ED = emergency department; HHS = U.S. Department of Health and Human Services; HRI = heat-related illness; NOAA = National Oceanic and Atmospheric Administration; NSSP = National Syndromic Surveillance Program; NWS = National Weather Service. * HeatRisk is a five-level, location-specific forecasting framework developed by NOAA/NWS and CDC that integrates meteorological data with human health metrics to assess and communicate the progressive, population-level risks for heat exposure in real time. HeatRisk was calculated using observed maximum and minimum temperatures from the NOAA Unrestricted Mesoanalysis at 2.5 sq km grid spacing, with values aggregated (median) to the county level for June 28–July 7, 2026. The highest HeatRisk category observed during this 10-day period was then mapped. HeatRisk | NWS; HeatRisk | CDC ^†^ To reduce artifactual impact from changes in reporting patterns, analyses were restricted to facilities with a coefficient of variation for ED visits ≤40 and average weekly informative discharge diagnosis completeness ≥70%, with discharge diagnosis code formatting consistent during January 1, 2018–September 10, 2026. After data quality filters were applied, a maximum of 1,069 EDs (range = 3–1,069; median = 39) that participate in NSSP returned one or more visits associated with HRI. ^§^ HHS Regional Offices | HHS.gov ^¶^ Puerto Rico and the U.S. Virgin Islands do not currently report data to NSSP.

## Preliminary Conclusions and Actions

The June 28–July 7, 2026, heat dome was one of several events during the 2026 heat season associated with increased HRI ED visit rates. During periods of extreme heat, persons in affected areas can monitor heat warnings and advisories and use early heat warning systems (e.g., HeatRisk) with adequate lead time to plan and prepare for outdoor activities. If home air conditioning is unavailable, persons can follow recommendations from local and state emergency agencies on ways to mitigate extreme heat exposure, including guidance on accessing cooling centers and other air-conditioned spaces. Public health agencies can continue to use near real-time heat-health information systems,^¶^ and clinical and public-facing guidance documents** (*3*) to detect, track, and communicate heat-related impacts.

## Footnotes

* Data quality filters were used to reduce artifactual impact from changes in reporting patterns. Specifically, for comparing visit rates across HHS regions and years, analyses were restricted to facilities with a coefficient of variation for ED visits ≤40 and average weekly informative discharge diagnosis completeness ≥70%, with discharge diagnosis code formatting consistent during January 1, 2018–September 10, 2026.

† 45 C.F.R. part 46.102(l)(2), 21 C.F.R. part 56; 42 U.S.C. Sect. 241(d); 5 U.S.C. Sect. 552a; 44 U.S.C. Sect. 3501 et seq.

§ All facilities reporting HRI data to NSSP were included in the total HRI ED visits reported during January 1–September 10, 2026, across all HHS regions. During this period, HRI data were reported by a maximum of 1,737 EDs (range = 23–1,737; median = 213) that participated in NSSP and returned one or more visits associated with HRI. For comparing visit rates across HHS regions and years, data quality filters were applied, resulting in a maximum of 1,069 ED facilities (range = 3–1,069; median = 39) that participated in NSSP returning one or more visits associated with HRI.

¶ For example, CDC’s Heat and Health Tracker is an interactive, online information system designed to help communities prepare for and respond to extreme heat. The tracker combines real-time weather forecasts, historical weather data, and ED visit records to assess local heat risks and protect populations at risk for HRI.

** Heat health resources: Heat.gov | National Integrated Heat Health Information System; Heat Health | CDC
